# On the Use of Assistive Technology during the COVID-19 Outbreak: Results and Lessons Learned from Pilot Studies

**DOI:** 10.3390/s22176631

**Published:** 2022-09-02

**Authors:** Laura Fiorini, Erika Rovini, Sergio Russo, Lara Toccafondi, Grazia D’Onofrio, Federica Gabriella Cornacchia Loizzo, Manuele Bonaccorsi, Francesco Giuliani, Gianna Vignani, Daniele Sancarlo, Antonio Greco, Filippo Cavallo

**Affiliations:** 1 Department of Industrial Engineering, University of Florence, 50139 Florence, FI, Italy; 2 The BioRobotics Institute, Scuola Superiore Sant’Anna, 56025 Pontedera, PI, Italy; 3 ICT, Innovation and Research Unit, Fondazione IRCCS Casa Sollievo della Sofferenza, 71013 San Giovanni Rotondo, FG, Italy; 4 Umana Persone Development & Research Social Enterprise, 58100 Grosseto, GR, Italy; 5 Clinical Psychology Service, Health Department, Fondazione IRCCS Casa Sollievo della Sofferenza, 71013 San Giovanni Rotondo, FG, Italy; 6 Co-Robotics S.r.l., Capannoli, 56125 Pisa, PI, Italy; 7 Geriatrics Unit, Department of Medical Sciences, Fondazione IRCCS Casa Sollievo della Sofferenza, 71013 San Giovanni Rotondo, FG, Italy

**Keywords:** assistive technology, social inclusion, telepresence, disinfection robot, remote monitoring, COVID-19 pandemic emergency

## Abstract

As a consequence of the COVID-19 emergency, frail citizens felt isolated because of social isolation, suspended and/or strongly reduced home assistance, and limited access to hospitals. In this sense, assistive technology could play a pivotal role in empowering frail older adults reducing their isolation, as well as in reinforcing the work of formal caregivers and professionals. In this context, the goal of this paper is to present four pilot studies—conducted from March 2020 to April 2021—to promptly react to COVID-19 by providing assistive technology solutions, aiming to (1) guarantee high-quality service to older adults in-home or in residential facility contexts, (2) promote social inclusion, and (3) reduce the virus transmission. In particular, four services, namely, telepresence service, remote monitoring service, virtual visit, and environmental disinfection, were designed, implemented, and tested in real environments involving 85 end-users to assess the user experience and/or preliminary assess the technical feasibility. The results underlined that all the proposed services were generally accepted by older adults and professionals. Additionally, the results remarked that the use of telepresence robots in private homes and residential facilities increased enjoyment reducing anxiety, whereas the monitoring service supported the clinicians in monitoring the discharged COVID-19 patients. It is also worth mentioning that two new services/products were developed to disinfect the environment and to allow virtual visits within the framework of a hospital information system. The virtual visits service offered the opportunity to expand the portfolio of hospital services. The main barriers were found in education, technology interoperability, and ethical/legal/privacy compliance. It is also worth mentioning the key role played by an appropriate design and customer needs analysis since not all assistive devices were designed for older persons.

## 1. Introduction

As of April 2021, according to World Health Organization (WHO) [1], more than 131 million people were affected and almost three million people died due to COVID-19 worldwide. In Europe, there were more than 46 million people affected by COVID-19, and, in Italy, almost 3.6 million people were affected by COVID-19, of which 107,569 people died. According to Istituto Superiore di Sanità (ISS) [2], in Italy, the median age of deceased COVID-19-positive patients was 81 years (median 82, range 0–109, interquartile range (IQR) 75–88) corresponding to 61.7% of the total. The number of deceased women was 46,852 (43.9%). There were 1188 (1.1%) deceased COVID-19-positive patients younger than 50 years of age.

Due to these health and safety countermeasures, global citizens faced unprecedented changes to their daily routines, including stay-at-home orders, travel bans, and closures of educational institutions and entertainment-related locales. Consequently, pandemic counteracts have had a significant psychological impact, mostly due to the social distancing, which has been the major cause of loneliness, especially in nursing or old-age homes [3]. It is also worth mentioning that, during the lockdown, in-home assistant services were reduced or suspended because of the restrictions [4]. Similarly, visits to hospitalized patients were also suspended. Additionally, it is well documented that COVID-19 restrictions increased the stress level of caregivers living with people suffering from dementia [5,6] or amyotrophic lateral sclerosis [7]. In this sense, new ways of performing social care and a new paradigm of clinical assistance should be introduced to face the problem, with the assurance of the same level of assistance to frail older adults [8]. Additionally, more emphasis and adequate solutions to the question “who cares?” with a multidisciplinary focus on the older adults in these times of pandemic is needed [9], since most of their needs have changed [10].

The 71st World Health Assembly (2018) recognized the critical contribution of assistive technologies to promoting inclusion and participation in all areas of society. Additionally, as remarked by the World Intellectual Property Organization [11], the changing market demographics for assistive technology, including the aging population, present opportunities to inventors and a potential change of paradigm in the market share. In this sense, assistive and digital technology (including robotics) could represent a new consolidated model of clinical practice in the management not only of COVID-19 but of the spread of infectious diseases in general [12,13]. In this sense, the COVID-19 emergency has caused an unprecedented boost toward the use of technology in different sectors, including clinical practice. According to the statistics on Italian citizens from 21 February 2020 to 12 April 2020, there was an 11% increase in the time spent on the internet and remote communication [14]. Statistics underline an increased use of Zoom, Google Classroom, and Microsoft Teams during the first half of March 2020 [15], as well as online payments [16] and online food ordering. Indeed, empirical studies have also reported an increasing trend of ICT use and a higher risk of excessive internet use during COVID-19 quarantine or lockdown [17].

According to the state of the art, the cooperation between assistive product/devices (in the literature, assistive products are defined as “*any product (including devices, equipment, instruments, and software) either especially designed and produced, or generally available, whose primary purpose is to maintain or improve an individual’s functioning and independence, thereby promoting their wellbeing*” [18]) and clinical staff could give rise to new prevention protocols that effectively and promptly counteract the spread, in the future, of infectious diseases capable of putting at risk the public health of our country and the globalized system in which we live, as remarked in [12,19]. For instance, during the COVID-19 pandemic emergency, a robot could be used mainly in high-risk areas of hospitals for dispensing meals and providing telehealth, thereby preventing human-to-human contact and reducing the potential of contagion [20,21,22]. Similarly, they can be used to support daily life during social distancing, promoting relationships between humans [23,24]. Additionally, robots can be employed for quarantine enforcement such as for the disinfection of public spaces by local government and public authorities [20]. Furthermore, according to literature evidence, assistive technology could support the extended caregivers’ ecosystem in multiple ways by leveraging their burden [10,19,25]. In this sense, it is essential and critical that public governments continue to work toward the development of sustainable policies that promote the use of assistive technology among frail citizens, disrupting the barriers at the entrance [21,26].

In this study, the COVID-19 emergency was the contextual predisposing factor for leading the design, development, and testing of assistive devices in different operative contexts. Indeed, this paper aims to present the experience of the use of assistive devices at four different pilot sites during the COVID-19 outbreak. We called this action “fast pilot”, referring to the need of implementing quick solutions able to provide an immediate reaction to the lack and/or interruption of the assistance services caused by the pandemic. Given previous research, lack of assistive device ownership and knowledge, as well as the digital divide, can lead to a poor user experience of technology. Therefore, to overcome this barrier, this study aimed to apply a five-step methodology that applied co-design principles to design, develop, and test efficient solutions driven by changing user needs. Conversely to other studies that were more focused on unique solutions (e.g., telepresence [27], disinfection [28], and monitoring [29,30]) or remote surveys [10,19], this paper presents four different services, namely, telepresence, virtual visit, remote monitoring, and environment disinfection services, developed on the basis of real user needs (presented in [10]). As for assistive technologies, a telepresence robot, a service robot, sensors, and web services were used, exploiting a different level of integration, among other issues. The tests were evaluated using qualitative and quantitative tools to point attention to the acceptance (as in [31,32,33]) and technology reliability among other factors. The collected results are aimed at providing the scientific community with guidelines and lessons learned to provide a holistic overview of the multidomain barriers encountered during the experience of testing different solutions in different operative scenarios, thus reducing the time to market for the actual adoption of the proposed services.

## 2. Material and Methods

**Figure 1:**
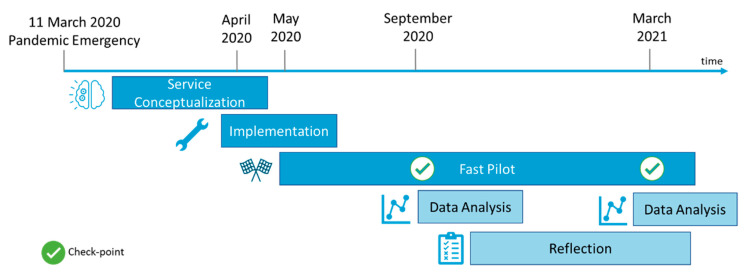
Phases of the proposed pilot study together with the timeline. Two checkpoints were fixed in September 2020 and in March 2021 to evaluate the proposed services.

The methodology applied in this paper is depicted in Figure 1 and follows five different phases, namely, (1) service conceptualization (Section 2.1), (2) implementation (Section 2.2), (3) fast pilot (Section 3), (4) data analysis (Section 3), and (5) reflection (Section 4). The sections below detail each phase of the methodology.

### 2.1. Phase 1: Service Conceptualization

**Figure 2:**
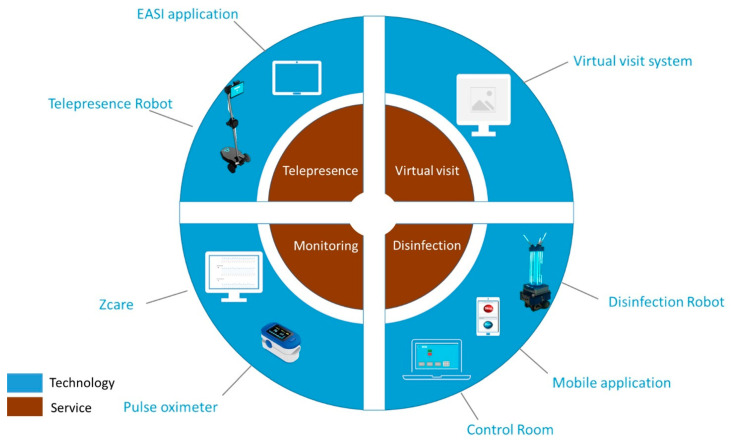
Overview of the assistive technology used within the proposed fast pilot. The telepresence service was performed with the robot and with the tablet. As for the telepresence service with the robot, two telepresence robots were used: Ohmni and Double robot.

The idea was conceived within the framework of the Italian pilot site of the Pharaon Project (Pharaon Project, official website: https://www.pharaon.eu/ (accessed on 20 July 2022)) (GA 857188) coordinated by the University of Florence (Scuola Superiore Sant’Anna was the coordinator until 31 October 2020). Other members of the Italian pilot actively involved in the proposed fast pilot were Umana Persone s.r.l., a network of 10 social cooperatives that operate in the Tuscany Region, the “Casa Sollievo della Sofferenza” Research Hospital, located in San Giovanni Rotondo (Apulia Region), and Co-Robotics s.r.l., a spin-off company of Scuola Superiore Sant’Anna with expertise in robotics. After the World Health Organization (WHO) declaration of the pandemic emergency on 11 March 2020, the members of the Pharaon (Pharaon Project, official website: https://www.pharaon.eu/ (accessed on 20 July 2022)). Italian pilot virtually met to discuss the use of assistive technology/device to support citizens, as well as the work of the professionals. The rationale was to propose a fast response to the emergency, conceptualizing solutions that could be implemented (and used) in real environments in less than 2 months. The 10 members of the Italian pilot involved in this meeting had different backgrounds (five engineers, one doctor, one head of innovation in the hospital, two social professionals, and one psychologist were involved in the focus group). They were requested to discuss the situation they were facing in the Tuscany territory and at the hospital using preliminary research outcomes presented in [10]. The aim of the focus group was twofold: (i) to define needs that were raised in social and clinical context so as to conceptualize services, and (ii) to identify potential assistive technologies that were already available in the pilot because of other projects (or donations) that could be used or adapted (and used) in the pilot in less than 2 months. On the basis of the highlighted needs, as discussed during the meeting and reported in Table 1, four services were outlined, namely, telepresence, virtual visit, remote monitoring, and environmental disinfection services (Figure 2).

### 2.2. Phase 2: Service Implementation

**Figure 3:**
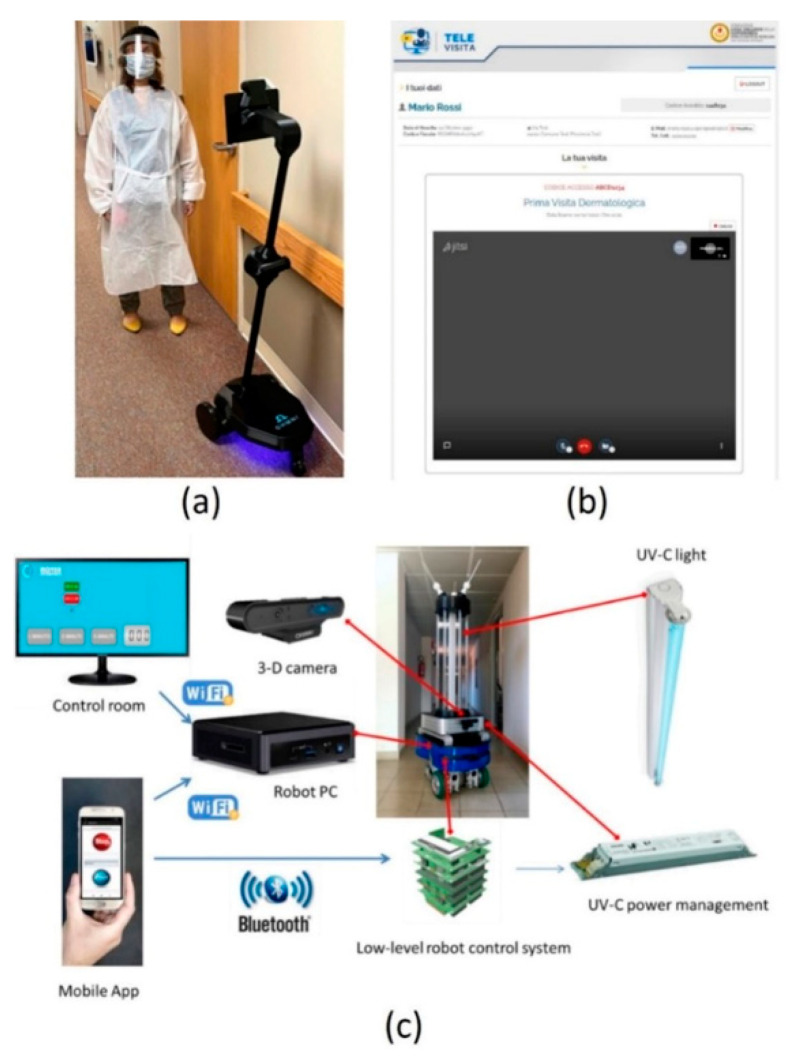
(**a**) Ohmni robot and professional caregiver during the telepresence service in a residential facility; (**b**) user interface developed for the virtual visit service; (**c**) disinfection robot and its main components.

This phase aimed to implement the identified assistive technology solutions (Figure 3) by adapting the technology already present in the Italian pilot to the conceptualized services (if necessary) [34] (Figure 2). Thanks to synergies with other regional and national projects (i.e., Pronto Badante, CloudIA, SI-Robotics, Samaritan, and Artes 4.0) or private donations (from Zucchetti, Fondazione Marmo, and DIA Vending s.r.l.), the members can count on assistive technologies that can be reused (as they are or with slight adaptations) to meet the changing needs due to the pandemic. The services were addressed using specific information technology equipment while considering the national and regional regulations on social distancing and the restriction on people’s access to the hospital. Specifically, according to the requested implementation complexity, three different levels of adaptation were identified: (i) low, no need for adaptation, technology used as it is; (ii) medium, slightly/moderate integration and/or software adaptation performed; (iii) high, new software and/or hardware modules integrated to perform the service. Details on the system development and the levels of adaptation (i.e., low, medium, and high) are described in Table 1.

### 2.3. Participants

The participants in the fast pilots (Phase 3) were enrolled from two Italian regions: Tuscany and Apulia. According to the services, the recruitments were conducted by the “Casa Sollievo della Sofferenza” Research Hospital (for patient remote monitoring and virtual visit services), by the Network of Social Cooperative Umana Persone (for telepresence service) and University of Florence and Co-Robotics (for disinfection service). According to the adaptation level (i.e., low, medium, and high, as described in Table 1), services were tested with end-users for a prolonged time in an unstructured environment or were tested in laboratory to assess the reliability of the system. Exclusion and inclusion criteria are reported in Section 3 for each service. One individual was enrolled to test only one service. All the tests were run in parallel between May 2020 and March 2021, as depicted in Figure 1 (fast pilot phase). Table 2 reports a summary of the number and type of participants recruited for each service.

## 3. The Fast Pilots

This section reports and describes the four pilot sites (Phase 3) in terms of the experimental setup, involved technology, and recruitment process; additionally, it briefly summarizes the results. According to the technology adaptation level, the tests were evaluated with different frameworks. Services characterized by a low adaptation level were tested in real environments focusing the efforts on assessing the technology acceptance after the use; on the contrary, services characterized by medium/high adaptation level were preliminarily tested in the laboratory environment to assess the technical feasibility and accuracy and preliminarily tested with end-users to assess the acceptance.

### 3.1. Telepresence Service

#### 3.1.1. Experimental Setup

There was no fixed time that the user was requested to interact with the device. Participants could continue to use it as long as they needed to. At the beginning and at the end of the trial, older adults were requested to compile an ad hoc questionnaire, to measure the usability and acceptability of the telepresence service as detailed in our previous work [35] (the questionnaire is provided in the Supplementary Materials). Additionally, a deep interview was conducted involving social managers to investigate the use, the experience, and the opportunities of using such technologies. The proposed fast pilot was tested in two operational scenarios:

- Private home—This scenario involved self-sufficient older people suspended from the usual home care service to reduce the risk of contagion. This scenario is important because it allows continuing to monitor the wellbeing and to keep active the aspects of socialization and support at a distance.
- Residential facility—This scenario involved COVID-19-infected (or suspected) older adults living in residential facilities without specific COVID-19 units. This scenario is important because it allows operators to monitor users’ conditions, especially at night, without physical presence.

#### 3.1.2. Recruitment

The recruitment was conducted within eight social cooperatives operating in the Tuscan territory as part of the network of “Umana Persone s.r.l.”. As for the inclusion criteria, we considered eligible for this study for the “private home” scenario all frail older adults (over 65 years old) already supported by domiciliary assistance, who were at risk of isolation because of COVID-19 restrictions. As for the residential facility scenario, we considered eligible for this study all suspected cases of COVID-19 that needed to be isolated. This study was approved by the Ethical Committee of Area Vasta Sud-Est (C.E.A.V.S.E.) Prot. n. 153 on 17 November 2020.

#### 3.1.3. Results

A total of 23 older adults and three professionals were enrolled for this service from the beginning of the pilot. Three older adults enrolled for the telepresence at home with the tablet were excluded from this report because of interruptions due to internet connection problems (two participants) and hospitalization. Additionally, one participant enrolled in the nursing home scenario did not complete the questionnaire at the end of the trial and, thus, was excluded from the analysis. The service is still running for three participants since there was no fixed end-time; hence, they were not included in this analysis. Finally, 16 participants were included in this study (10 older adults used the Ohmni robot in the residential facility, four older adults used the tablet in private homes, and two older adults used the Double robot in private homes). As for the average length of the trial computed as the difference in days between the start day (T0) and the end of the trial (TF) for each participant, in the residential facility, the robot was used for 14.9 days—comparable to the required quarantine duration, while it was used at home for 149 days; the tablet was used for 73 days.

**Figure 4:**
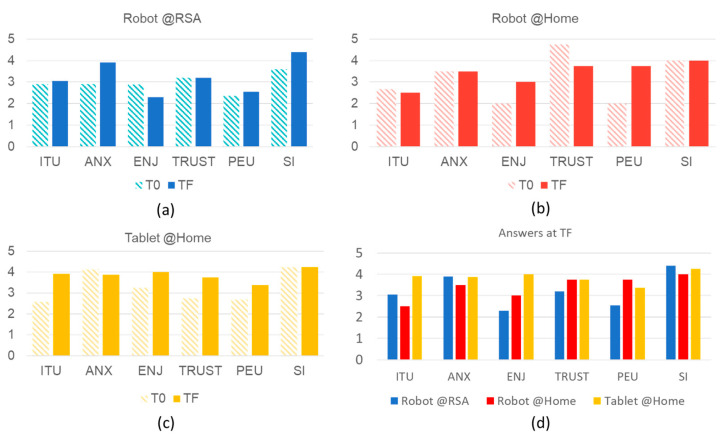
Results of the questionnaire for each construct at the beginning of the trial (T0) and at the end of the trial (TF) for the telepresence service: (**a**) average results of the telepresence service conducted with the robot in the residential facility; (**b**) average results of the telepresence service conducted with the robot in a private home; (**c**) average results of the telepresence service conducted with the tablet and EASI application in a private home; (**d**) comparison of the average results over the three applications at TF. ITU (intention to use); ANX (anxiety); ENJ (enjoyment); PEU (perceived ease of use); SI (social influence).

As for the questionnaires, the Crombach alpha coefficients (α) were computed for the questionnaires collected at the beginning and at the end of the trial to assess the reliability of the answers. The questionnaires are reliable (α initial = 0.88, α end = 0.84) and, therefore, further analyzed. All negative items (anxiety (ANX) and social influence (SI) items) were inverted to be easily compared and added to the analysis. Specifically, the average values were computed at T0 and TF (Figure 4). By observing the results obtained in the residential facility, it was noticed that the ANX and SI domains increased after robot use, which suggests that the user expected to feel more anxious and to feel more social judgment before the use. On the other hand, within the in-home trial, the enjoyment (ENJ) and the perceived ease of use (PEU) increased. These results suggest that the robot was expected to be more difficult to use before the trial, as in [35]. As for the use of the tablet, four out of six domains increased after long-term use. All these results suggest that older adults often had a prejudice about the use of assistive technology. The comparison of the results at TF (Figure 4d) underlines that, at home, the robot was rated better than the tablet in terms of PEU, while all participants rated the TRUST as high even if they rated the ENJ differently. The ENJ such as the general attitude during the trial was remarked also by the social operators during the interview (see Section 3.1).

### 3.2. Patient Remote Monitoring

#### 3.2.1. Experimental Setup

Patients were proposed to measure oxygen saturation and heart rate through pulse oximeter equipment, as well as blood pressure and body temperature. All these parameters are good indicators of the physical health status of the patients who faced the COVID-19 virus (risk score). Moreover, a survey on personal health conditions was compiled daily. Performed measurements and compiled surveys were registered by patients on a form through the ZCare web platform (designed by Zucchetti) or through a phone call to the professional caregiver, possibly several times a day, at any time. Physicians and caregivers, while offline, were then able to check the registered measurements through a dashboard, the answers to the survey, and the computed risk scores, thus enabling intervention if needed.

#### 3.2.2. Recruitment

Discharged COVID-19 patients by the CSS Geriatrics Unit in the period March–June 2020 were recruited voluntarily. Inclusion criteria for this pilot study were (i) informed consent for the participation in the study, (ii) absence of physical and cognitive frailties that may have prevented the compilation of the web form registry, and (iii) availability at the patient’s house of a personal computer and an internet connection. The system was compliant with the GDPR requirements. Specific consent was signed by the patient about the use and management of their data, and a specific privacy impact assessment was performed before starting the pilot.

#### 3.2.3. Results

A total of 32 patients discharged from the Geriatrics Unit were recruited until September 2020; 53% of the patients were female, with an average age of 72.3 ± 5.4 years old. The average duration of the hospitalization was of 20.1 ± 3.4 days. The average follow-up period was 14 ± 3.2 days with an average severity score of 3.1 ± 0.8. The average severity score showed a significant variation between the first and the 14th day of de-hospitalization: 3.6 ± 0.3 vs. 2.6 ± 0.2 (*p* < 0.05), confirming a clinical improvement. Fortunately, no patient needed re-hospitalization or any intervention by professional caregivers. All patients reported that they felt safer thanks to the monitoring after the hospital discharge. Collected data in the monitoring pilot study were, thus, available for further study and analysis of the recovery and the effects of the infection during the de-hospitalization phase.

### 3.3. Virtual Visit

Due to the COVID-19 restriction, hospitals had to reduce and, in some cases, completely stop public access to their facilities, thus reducing the availability of outpatient services. Some of the outpatient services could be delivered remotely by healthcare professionals through ICT systems designed to enable both patients and caregivers in performing virtual outpatient services as if they were delivered on site. All eHealth processes had to be compliant with the Italian Telemedicine Guidelines (“Linee di indirizzo nazionali sulla telemedicina, 2011”) which were approved by the Italian Conferenza Stato-Regioni in 2014 (Guidelines from Italian State-Region Conference “Conferenza Stato-Regioni: http://www.regioni.it/cms/file/Image/upload/2014/5_SR_20022014.pdf” (accessed on 2 July 2022)). The implementation of the guidelines is a warranty of fulfillment of ethical standards in terms of privacy, dignity, and quality of the medical act.

#### 3.3.1. Implementation

After an analysis of the as-is on-site outpatient services and processes, medical prescriptions had to be digitalized, and an upgrade of the internal hospital information system had to be performed to support digitalized prescriptions and online reservations. Afterward, the first phase of training sessions for the qualification of healthcare professionals and collaborators at the central appointment service (CUP) unit was planned. The other on-site outpatient services were not affected by the introduction of virtual visit services. An evaluation of the available videoconferencing technologies was required, and security aspects were taken into consideration, alongside privacy aspects and general data protection regulation measures. The service was preliminarily tested with end-users to ensure technical reliability. In parallel, CSS tried to ensure compliance with the ethical standard, as well as integration with the CUP (enterprise reservation system) and EMR to foster the adoption by patients and caregivers.

#### 3.3.2. Results

Despite the system being fully functional and preliminarily tested at CSS by five individuals, it is waiting for regional regulation (delibera regionale) that will legitimize and provide due reimbursement for the use of the virtual visit service in Apulia region in agreement with the national rules on telemedicine services (Indicazioni nazionali per l’erogazione di prestazioni in telemedicina) issued in December 2020 (Italian national regulation for telemedicine: http://www.statoregioni.it/media/3221/p-3-csr-rep-n-215-17dic2020.pdf (accessed on 2 July 2022)). Additionally, an online course to educate health care professionals on the use of telemedicine service was designed by CSS. The course has so far involved the participation of more than 150 healthcare professionals who have received the CME (crediti formativi) recognized by the Healthcare Education Commission of the Italian Ministry of Health.

### 3.4. Environment Disinfection

#### 3.4.1. Experimental Setup

The proposed service was evaluated within two main phases. In the first phase, the efficiency of the robot disinfection was tested in the laboratory setting (international standard ASTM E3135-18 was applied), whereas, in the second phase, it was tested in a real environment within the hospital “Fondazione Monasterio” located in Massa (IT). Additionally, an online survey was organized to collect feedback on robot acceptance through the selected construct of the Almere questionnaire [36] (see Supplementary Materials). The respondents were requested to watch the service demonstration (Demostration video of MoVer with UV-C lamp: https://www.youtube.com/watch?v=qP4JjAzFyfw (accessed on 15 July 2022)) and to rate each item on a five-point Likert scale, where 1 is “completely disagree” and 5 is “completely agree”.

#### 3.4.2. Recruitment

The questionnaire was disseminated using the internal hospital mailing list to involve professionals as a “potential” target group. An informed consent sheet was enclosed in the online questionnaire, which the participant accepted upon clicking. Feedback was also collected through interviews with nurses and doctors that were working in the hospital and saw the robot cleaning the room.

#### 3.4.3. Results

As for the efficiency test in the laboratory setting, two materials were selected (i.e., ISI 316 steel and polycarbonate) with three exposure times (i.e., 1, 3, and 5 min). The results underline an abatement percentage of >96% of bacteria for both materials after 3 min of exposure. The tests in the real environment were conducted considering the robot stationary in two positions, as well as while moving. The radiation doses were measured at key points of the room (e.g., bed, wardrobe, desk, and chairs) using a radiometer with a UVGI sensor (RMD Pro, Opsytec Dr. Gröbel, DE). The results underlined an improvement of the measured dose with the robot in movement with respect to the static test, suggesting that using a mobile robot could increase the efficiency for disinfection purposes.

As for the acceptance of the service, 22 participants (50% female) completed the online questionnaire. Cronbach’s alpha was computed to assess the reliability of the questionnaire; it was equal to 0.67, which can be considered quite reliable. As for the age, 37.8% of respondents were below 30 years old, 27.3% were between 31 and 45 years old, 31.8% were between 46 and 60 years old, and 9.1% were over 60 years old. Furthermore, 36.4% of respondents worked in the social assistance field, 22.7% worked in the ICT innovation field, and the remainder had different backgrounds. The results of the Almere questionnaire are reported in the Supplementary Materials; negative items were inverted. The results highlighted a general good acceptance of the proposed robot. It is worth mentioning that the perceived enjoyment was rated 4.14 out of 5, which means that almost all the respondents (mode = 5) thought that they would enjoy using the robot in their work. As for the other constructs, they were all rated higher than 3.50, with a mode equal to 4. Additionally, the robot was also certified as compliant with ISO 13482:2014 as a type 1.2 servant robot and compliant with ISO 10218 as an industrial AGV for use in domiciliary and industrial contexts.

## 4. Discussion: Lessons Learned from the Fast Pilots

**Figure 5:**
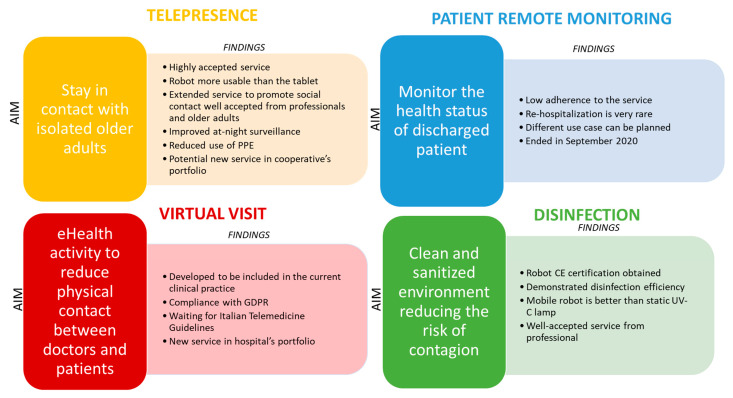
Lessons learned for each implemented service. For each service, the aims and findings are outlined.

This section discusses the results of each fast pilot, and Figure 5 summarizes the main contents. Additionally, Section 4.5 discusses the entire experience of designing, developing, and testing different services, while providing guidelines for further development.

### 4.1. Lessons Learned from the Telepresence Service

The service was highly accepted by social professionals and older adults, and the fast pilot is still active. According to the results collected (see Figure 4), within the domestic environment, the telepresence robot was considered more usable (PEU) than the tablet, and older adults enjoyed using the technology (ENJ) more than expected. This is aligned with the quantitative results of the questionnaire. Indeed, the robot was activated by the caregiver before interacting with the older adult. On the other hand, the case managers identified some functional and nonfunctional requirements for improving the usability of the application installed on the tablet. The robot used at the nursing home was rated as the lowest experience in terms of ENJ, TRUST, and PEU, because, as remarked by the social operators, the older adults that were isolated were generally in a bad mood and not open or prone to accepting anything. Nevertheless, it is worth mentioning, that, at the end of the experience, they felt less anxious about using the robot.

According to the feedback collected during the fast pilot, the use of a robot in this context is a more positive and acceptable service from the social operators’ point of view. Indeed, the proposed service created value, extending the assistance offered by the social cooperatives while proposing a service to promote virtual social contacts. The debriefing interviews with three social managers underlined that, within the nursing home, the robots were used to monitor the “potential” COVID-19 guests that were isolated from the others, especially at night, to improve surveillance. This allowed the caregiver to move into the “forbidden” zone with the robot and check the status of the guest, thereby saving time and avoiding the adoption of personal protective equipment (PPE) while guaranteeing a high level of personal security and saving related costs. As for the robot, two different telepresence robots were used. Caregivers preferred the Ohmni robot over the Double robot since it had more stability and was easier to drive.

### 4.2. Lessons Learned from the Virtual Visit Service

The COVID-19 emergency has accelerated the development of the virtual visit service, which has been implemented in the custom portfolio of the enterprise services offered by “Casa Sollievo della Sofferenza” Research Hospital. This will generate business value because it extends the healthcare offer of hospital care services. The development of the service was facilitated by the availability of systems that are mature in terms of compliance with the GDPR. Additionally, it was developed to be included in the current clinical practice. There was a high level of acceptance of the virtual visit by patients and physicians, specifically when this was the only way to perform visits given the restrictions stemming from the COVID-19 constraints. December 2020 was an important breakpoint for the development of telemedicine service in Italy, when the guidelines were released.

### 4.3. Lessons Learned from the Monitoring Scenario

All patients reported that they felt safer thanks to the monitoring after the hospital discharge. Caregivers and healthcare professionals were then able to quickly supervise the recruited patients while “offline” thanks to the web platform. It is also worth remarking that a lower adherence was registered after the first few days of use of the system. This is because the symptoms were extinguishing after hospital discharge; thus, patients had nothing of anomalous to register. This fast pilot ended in September, after the first wave of COVID-19, because the degree of monitoring system usage did not meet the expectations set when it was introduced by the hospital staff and because of the low adherence. The main reason is that, at the beginning of the emergency, healthcare professionals were not fully aware of the length of the period needed for post-COVID-19 symptoms to disappear. Fortunately, no patient needed re-hospitalization or any intervention by professional caregivers; therefore, the service was not perceived as a necessity during the second wave of the pandemic. Nevertheless, thanks to the system, CSS was able to confirm the impression of healthcare professionals that, after the discharge of the patient, a new hospitalization admission was very rare.

### 4.4. Lessons Learned from the Disinfection Scenario

Thanks to the fast pilot, the efficacy of the disinfection function was demonstrated in a relevant environment, and the obtained certification generates business value. Indeed, the tests demonstrated that the robot with navigation ability was more efficient in cleaning compared to a static UV-C lamp. The pandemic emergency accelerated the process of developing robots able to kill bacteria and viruses, thus improving security at work. The developed robot can be used in different environments such as hospitals, gyms, nursing homes, schools, and hotels. From the usability point of view, the system should include an automatic adaptation of cleaning procedures since they are dependent on the environment. The robot was well accepted, as confirmed by the outcome of the questionnaire.

### 4.5. Final Remarks and Future Developments

The presented fast pilots gave the unique opportunity to test the solutions based on assistive technology in relevant environments so as to collect information and experience related to the use in an emergency context. The proposed services make use of assistive technology with different technology readiness levels (TRLs). The solutions were developed starting from consolidated (i.e., commercial) assistive technologies that were used as they are (as in the telepresence service), slightly adapted (as in the remote monitoring scenario), or integrated (as in the sanitizing environment) to address the needs. In particular, the telepresence service used commercial robots (namely Double robot and Ohmni robot) in a different context to which they were marketed. Within the monitoring service, a customized web application was developed, whereas, for the virtual visit service, Jitsi was adapted and integrated within the internal hospital information system. Lastly, within the disinfection service, a new robot with an embedded UV-C lamp was proposed. In order to provide efficient and “fast” solutions, one important thing to consider is the **interoperability** among different devices, platforms, and vendors, since it is easier to build technology if it can be integrated into an already used platform. For instance, the virtual visit service was integrated into the hospital information system. This was applied as “Casa Sollievo della Sofferenza” Research Hospital saw the **potential business value** in offering telemedicine services through the video visit system, effectively deciding to maintain it as a stable service in its portfolio. From the beginning of this service conceptualization to the final test, the developers realized a service that was fully integrated in the current practice and the infrastructure/management system, thus encouraging its adoption in clinical care.

It is worth underlining that three out of the four services are still active as of March 2021. This achievement remarks the importance of correctly identifying the needs of customers in the service definition, as well as involving highly reliable and acceptable technologies, as also remarked by Gao et al. [25]. Five older adults are still using the tablet at home within the telepresence service, while social operators working at the nursing home activate the telepresence robot as soon as they have a possible COVID-19-positive guest to check their status at night. It is important to highlight that the **attitude of the caregiver** toward the assistive technology made a difference in the effective use of the technology, as also underlined by Smith et al. [19].

The pilots related to telepresence service showed that providing assistive devices as along with internet, instruction, and training changed the perception of the technology. Indeed, we can observe a positive trend in Figure 4. These results are aligned with other studies conducted during the pandemic that underlined an increasing use of telecare services during the COVID-19 outbreak [37] and a large-scale survey that underlined the positive use of robots in some telecare tasks [38]. To summarize, **people need to be educated** on the use of technology. This training should cover not only basic digitalization (e.g., internet access and use of the computer) but also the potential use of the digital tools in their daily routine. This can be potentially achieved by introducing technology at the academic level in non-technical/engineering courses (e.g., for nurses and doctors). The importance of the educational aspect was also underlined by Deloitte [39] as one of the primary barriers to technology adoption in Italy. The remote monitoring service was interrupted after the first wave of the pandemic since the professionals noticed a reduced service adherence. Nevertheless, the same technology can be used with COVID-19-positive people at risk of getting worse to monitor their physiological status and, therefore, promptly intervene if necessary. Table 3 summarizes and highlights the multidisciplinary guidelines that we learned from the operative fields.

## 5. Conclusions

This paper presented four fast pilots aimed at empowering and supporting older adults and professionals during the pandemic emergency. The services were conceptualized and modulated on the needs perceived by the social operators and the clinical professionals involved during the early stage of the COVID-19 emergency (March–April 2020).

Indeed, the main needs addressed were (i) to improve the social connection/inclusion of older adults living at home since the in-home services were suspended, (ii) to limit the risk of contagion at work by providing the disinfection robot service and the telepresence service to remotely check the status of isolated older adults living in a nursing home, (iii) to monitor the discharged COVID-19 patients at home, and (iv) to assist older adults and frail citizens by remotely offering psychological support or follow-up visits through video sessions. This paper presents preliminary findings and lessons learned from the experience and real use of the technology in the emergency context. It is also worth mentioning that three out of four services after 1 year are still ongoing, and this result underlines the utility of the implemented services during the pandemic. These services, indeed, have been able to create economic value, promoting their business exploitation among the partners involved in this work. The hospital is interested in adding the virtual visit service to its clinical service portfolio, and the social cooperatives are interested in using the telepresence service in their home assistance services across the territory.

Despite the high acceptance of the services and the declared necessity of telehealth and telecare systems, a digital divide persists. Indeed, “education” is one of the key barriers that was identified at the end of this work. This educational barrier is defined in broader terms as a lack of information on and ownership of communication technology devices (e.g., cell phones, laptop computers, and tablets), internet access, and ICT know-how, while it also includes some biases that older adults and caregivers can exhibit in front of new technology. We need to change our mentality and attitude toward technology; indeed, in our fast pilot we provided evidence that the use of technology could “change” the domain of acceptance. In other terms, if we provide the right support, we can overcome the bias that we may encounter. Lastly, important lessons learned through these actions enabled suggesting issues and guidelines that researchers and companies have to face and address for favoring the implementation of such services in other realities and use cases. Further studies, with larger sample sizes, should be conducted to overcome the limitations of the present work.

## Figures and Tables

**Table 1:** Relationship between the needs identified and the implemented services.

Identified Needs to Be Addressed	Service Description	Technology	Level of Technology Adaptation ^a^
Improving the social contact with an isolated older adult when there are restrictions related to personal contact that could suspend traditional in-home assistance and outpatient visits, as well as limit the services in residential care.	**Telepresence Service**—Due to COVID-19 restrictions, in-home social services have been suspended and/or reduced. Within residential facilities, people suspected to be affected by COVID-19 (i.e., awaiting swab results) stay isolated from the other guests. Nevertheless, using the telepresence system, social operators can talk in total “safety” with people several times a day, thus increasing the number of visits and reducing social isolation.	**Double robot** (official website: https://www.doublerobotics.com/ (accessed on 15 July 2022)) (Double robotics, USA) and **Ohmni robot** (Ohmnilab robotics, CA, USA) (official website: https://ohmnilabs.com/ (accessed on 15 July 2022)) were applied with proprietary user interfaces. Alternatively, tablets with **EASI android application** from Pronto Badante project were used.	**Low**
Checking the clinical status of COVID-19 patients after hospital discharge since the long-term effects of the infection are not fully clear. Therefore, physicians have manifested the need for specific scores to make de-hospitalization safer (e.g., the absence of fever for 48 h after discharge, improvement of the respiratory picture, and laboratory parameters).	**Patient Monitoring**—Thanks to the patient remote monitoring service, healthcare professionals can monitor patients after a hospital stay due to COVID-19 infection. Through a customized web page, they have to answer a set of standard questions related to clinical aspects (e.g., the presence of cough, headache, or anosmia) relevant in the case of COVID-19. Additionally, they have to monitor body temperature and blood oxygenation using two medical devices.	The system is composed of **ZCare**, a web platform designed to gather health data through a graphic user web interface, and to compute specific risk scores, alongside the use of **pulse oximeter devices** (Onyx Vantage Blue, model no. 9590, a medical device from Nonin Medical, Inc.). The system can automatically alert healthcare professionals in case of a risk score above a predefined threshold.	**Medium**
Reducing physical contact between doctors and patients and between patients in the waiting areas or registration desks in compliance with the prescription of the COVID-19 restriction.	**Virtual Visits**—This service allows performing a remote patient visit, i.e., a video visit session between doctors and patients. A dedicated service takes care of managing the contacts with the patients and planning the video visit sessions. The patient performs the administrative registration (including the payment) of the visit in the dedicated web portal. The clinical documentation is available in the hospital’s electronic medical record or is securely transmitted before the virtual video session. All medical prescriptions (clinical reports, drugs, etc.) that result from the virtual visit session are made available as digitally signed documents. The hospital aims to start an eHealth process that will be used in the future routinely, even beyond the current status of emergency determined by COVID-19	The system to perform virtual visits between the physician and the patient has been fully developed. It consists of a core application based on Jitsi (a free and open-source multiplatform VoIP and videoconferencing web application) that was adapted to be integrated with the enterprise hospital information system and specifically with the electronic medical records (EMR) solution. A sister system was developed to allow for the secure transmission of medical documentation.	**High**
Reducing virus transmission through cleaning and disinfection of environments, surfaces, and objects, thus guaranteeing safety in the workplace.	**Disinfection**—The purpose of this scenario was to develop a mobile robot able to disinfect and sterilize a hospital or nursing home room efficiently and smartly, using the germicidal capabilities of the UV-C lamps and the autonomous navigation of the robotic platform. The main idea is to have a robot that can autonomously move inside a room to reach all the corners and surfaces, to obtain better and faster sanitization.	The system is composed of **MoVer1** (Co-Robotics, Italy) as a mobile robotic platform equipped with **a set of ultraviolet C radiation lamps**. MoVer1 has an embedded computer and several sensors such as encoders, inertial measurement units, laser scanner, and depth camera. The robot mounts a set of nine low-pressure germicidal mercury lamps that emit ultraviolet C radiation with a 254 nm dominant wavelength. Specifically, six lamps (Philips TUV T8 tubular lamp) are mounted vertically along the central axis of the robot, and three lamps (OSRAM Puritech HNS G23) are mounted inclined at 45° on top of the first one, with this arrangement the irradiation power is increased considerably. Thanks to two user interfaces (i.e., both web-based and Android app-based), the operator can easily use the robot to disinfect the environment. Demostration video of MoVer with UV-C lamp: https://www.youtube.com/watch?v=qP4JjAzFyfw (accessed on 15 July 2022).	**High**

^a^ Technology adaptation level: low, no need of adaptation, technology used as it is; medium, slightly/moderate integration and/or software adaptation performed; high, new software and/or hardware modules integrated to perform the service.

**Table 2:** Summary of the recruited participants in each pilot site.

Service	Number of Citizens Recruited	Age	Number of Professionals Recruited	Location	Type of Test	Measures
**Telepresence**	23	Over 65 years old	3 ^a^	Tuscany	Use in real environment	Acceptance and duration
**Patient Remote Monitoring**	32	72.3 ± 5.4 years old	1 ^b^	Apulia	Use in real environment	Adherence and use of service
**Virtual Visits**	-	-	5	Apulia	Laboratory test	System feasibility
**Disinfection**	-	-	22	Tuscany	Laboratory test	System reliability and acceptance

^a^ Social operators that virtually met the older adults; ^b^ professional caregiver that accessed the monitoring scenario portal from the hospital.

**Table 3:** Highlights from the experience: results of the reflection phase.

Highlights from Lesson Learnt	Description
**Correct identification of the needs of customers**	It is important to correctly identify the needs of the users (i.e., the marked customer segment) and, thus, develop a service that is acceptable to the users. Indeed, to reduce the time to market, iterative co-creation or a user-centered design approach should be applied.
**Easy-to-use Technology**	The technology should be designed considering the final target users and make the service usable for them. In the telepresence service, the robot was considered more usable by older adults because they did not have to perform complex interactions with the user interface.
**Multidisciplinary evaluation of the services**	A service based on assistive technology needs to be evaluated from a multidisciplinary point of view since the final adoption is the result of the action of multiple factors (i.e., usability of the technology, reliability, cost–benefit relationship, integration with the current procedures, and impact).
**Education**	The attitude of the caregiver makes the difference in the efficacy of the pilot and the recruitment. People need to be educated and trained on the use of digital and assistive technology. Appropriated strategies to involve and educate the caregiver should be considered.
**Interoperable platform**	Assistive services should also be integrated into the current practice from a technical point of view. Indeed, as shown by the remote monitoring system, integrating the system into the existent clinical platform and IT systems favors its use. Future systems should be developed considering the interoperability issue, and they should be ready to be integrated into the used platforms.
**Ethical, legal, and GDPR compliance**	Privacy, ethical, and legal issues are important points to be considered. Therefore, when developing a new service based on assistive technology, it should be compliant with GDPR policies and guarantee data security and management.

## Data Availability

Data are not available.

## Supplementary Materials

The following supporting information can be downloaded at: https://www.mdpi.com/article/10.3390/s22176631/s1, Table S1: Constructs and Items of the questionnaire used for the telepresence service. Participants were asked to rate each construct on a 5 points Likert Scale; Table S2: ALMERE selected construct and items and results of the online questionnaire administrated for the disinfection service. Participants were asked to rate each construct on a 5 points Likert Scale.
