# Supplementary material for: On the Use of Assistive Technology during the COVID-19 Outbreak: Results and Lessons Learned from Pilot Studies
Source: Sensors (Basel). 2022 Sep 2;22(17):6631. doi: 10.3390/s22176631 (PMC9460223; doi:10.3390/s22176631)
Supplement: Supplementary file 1 [file sensors-22-06631-s001.zip › sensors-1854347-supplementary.pdf]

Table S1 . Constructs and Items of the questionnaire used for the telepresence service. Participants were asked to rate each construct on a 5 points Likert Scale.

Acronym	Construct	Item ("I think that…")<br>¥=robot or tablet
ITU	Intention of use	I would use ¥ in case of necessity<br>I would use ¥ in case my family need it<br>¥ will increase my independence
ANX	Anxiety	I feel nervous using ¥<br>¥ is too invasive for my privacy
ENJ	Enjoyment	I will enjoy using the ¥
TRUST	Trust	I would trust the ¥ if it gave me advice
PEU	Perceived ease of use	the ¥ is ease of use<br>I find easily the button to stop the service<br>The interface is ease of use<br>Talk to the caregiver through ¥ is easy
SI	Social Influence	I feel embarrassed to use ¥

Table S2. ALMERE selected construct and items and results of the online questionnaire administrated for the disinfection service. Participants were asked to rate each construct on a 5 points Likert Scale.

Acronym	Construct	Item ("I think that…")	Mean	SD	Mode
ANX	Anxiety	The robot scares me<br>The robot makes me anxious	3.50	1.07	4.00
ITU	Intention of use	I would often use the robot in my job	3.73	1.03	4.00
PU	Perceived usability	The robot could support me<br>It will be convenient for me to use the robot in my job	3.66	1.06	4.00
PEOU	Perceived ease of use	The robot is easy to use<br>I would need a person that helps me with the use<br>Persons could easily learn to use it	3.65	0.63	3.67
PENJ	Perceived enjoyment	I will enjoy using the robot	4.14	0.83	5.00
SI	Social Influence	I would give a good impression if I'll use the robot<br>Colleagues would like me to use the robot	3.64	1.13	3.00
TRUST	Trust	I would trust the robot if it gave me advice<br>I would follow the advice the robot gives me	3.73	0.91	4.00
